# Bis{2-meth­oxy-6-[tris­(hydroxy­meth­yl)methyl­imino­meth­yl]phenolato-κ^3^ *O*,*N*,*O*′}manganese(II) dimethanol solvate hemihydrate

**DOI:** 10.1107/S1600536809005364

**Published:** 2009-02-21

**Authors:** Xiutang Zhang, Peihai Wei, Jianmin Dou, Bin Li, Bo Hu

**Affiliations:** a Advanced Material Institute of Research, Department of Chemistry and Chemical Engineering, Shandong Institute of Education, Jinan 250013, People’s Republic of China; b College of Chemistry and Chemical Engineering, Liaocheng University, Liaocheng 252059, People’s Republic of China; c Department of Chemistry and Chemical Engineering, Shandong Institute of Education, Jinan 250013, People’s Republic of China

## Abstract

In the title complex, [Mn(C_12_H_16_NO_5_)_2_]·2CH_3_OH·0.5H_2_O, the Mn^II^ atom has a distorted octa­hedral coordination geometry in which two N atoms from two 6-meth­oxy-2-[tris­(hydroxy­meth­yl)methyl­imino­meth­yl]phenolate ligands adopt a *trans* arrangement. The Mn—O(H) bonds (mean length 2.134 Å) are significantly longer than the Mn—O and Mn—N bonds (mean length 2.011 and 2.027 Å, respectively), and the dihedral angle between the mean planes through the aromatic rings of the two ligands is 76.8 (1)°. A complex network of O—H⋯O hydrogen bonds is formed between the complexes and the uncoordinated methanol and water mol­ecules. The C and O atoms of one C—OH group are disordered with equal occupancies.

## Related literature

For Schiff-base complexes of transition metals, see: Ward (2007 ▶). For details of the synthesis and a related structure, see: Wang *et al.* (2007 ▶).
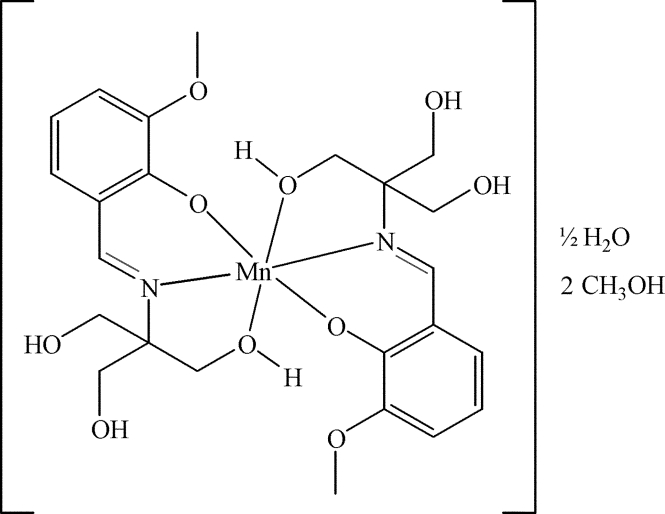


## Experimental

### 

#### Crystal data

[Mn(C_12_H_16_NO_5_)_2_]·2CH_4_O·0.5H_2_O
*M* *_r_* = 636.55
Monoclinic, 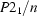

*a* = 8.141 (2) Å
*b* = 18.130 (5) Å
*c* = 20.211 (6) Å
β = 93.590 (4)°
*V* = 2977.2 (14) Å^3^
*Z* = 4
Mo *K*α radiation
μ = 0.51 mm^−1^
*T* = 293 K
0.12 × 0.10 × 0.09 mm

#### Data collection

Bruker APEXII CCD area-detector diffractometer
Absorption correction: multi-scan (*SADABS*; Sheldrick, 2003 ▶) *T*_min_ = 0.941, *T*_max_ = 0.955
14576 measured reflections
5287 independent reflections
4036 reflections with *I* > 2σ(*I*)
*R*_int_ = 0.031

#### Refinement

*R*[*F* ^2^ > 2σ(*F* ^2^)] = 0.051
*wR*(*F* ^2^) = 0.148
*S* = 1.05
5287 reflections
416 parameters
16 restraints
H atoms treated by a mixture of independent and constrained refinement
Δρ_max_ = 0.93 e Å^−3^
Δρ_min_ = −0.65 e Å^−3^

### 

Data collection: *APEX2* (Bruker, 2004 ▶); cell refinement: *SAINT-Plus* (Bruker, 2001 ▶); data reduction: *SAINT-Plus*; program(s) used to solve structure: *SHELXS97* (Sheldrick, 2008 ▶); program(s) used to refine structure: *SHELXL97* (Sheldrick, 2008 ▶); molecular graphics: *SHELXTL* (Sheldrick, 2008 ▶); software used to prepare material for publication: *SHELXTL*.

## Supplementary Material

Crystal structure: contains datablocks global, I. DOI: 10.1107/S1600536809005364/bi2342sup1.cif

Structure factors: contains datablocks I. DOI: 10.1107/S1600536809005364/bi2342Isup2.hkl

Additional supplementary materials:  crystallographic information; 3D view; checkCIF report

## Figures and Tables

**Table 1:** Hydrogen-bond geometry (Å, °)

*D*—H⋯*A*	*D*—H	H⋯*A*	*D*⋯*A*	*D*—H⋯*A*
O3—H3⋯O8	0.86 (1)	1.885 (10)	2.706 (5)	160 (4)
O4—H4⋯O9	0.86 (1)	1.802 (12)	2.664 (5)	174 (5)
O5—H5⋯O2^i^	0.85 (1)	1.886 (13)	2.736 (4)	173 (6)
O6—H6⋯O3	0.86 (1)	1.886 (9)	2.737 (7)	172 (6)
O9—H9⋯O11	0.85 (1)	1.888 (10)	2.712 (5)	162 (4)
O10—H10⋯O1*W*	0.86 (1)	1.90 (4)	2.585 (6)	136 (5)
O11—H11⋯O6^ii^	0.85 (1)	1.897 (18)	2.739 (4)	169 (7)
O12—H12⋯O2	0.85	2.12	2.967 (7)	180
O12*A*—H12*A*⋯O5^iii^	0.85	2.10	2.946 (7)	180
O1*W*—H1*W*⋯O5	0.85	1.81	2.657 (8)	180
O1*W*—H2*W*⋯O12*A*^iv^	0.85	2.14	2.990 (9)	180

Symmetry codes: (i) 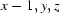; (ii) 
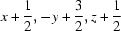
; (iii) 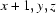; (iv) 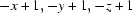.

**Fig. 1.**
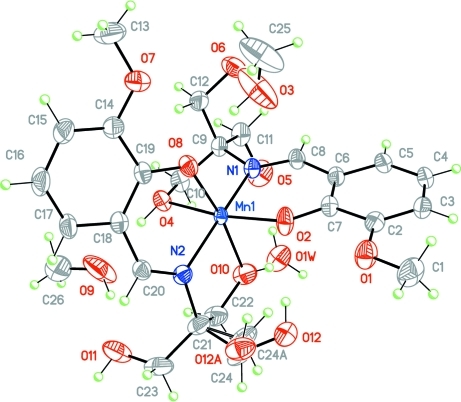
The molecular structure with displacement ellipsoids at 30% probability for non-H atoms.

## supplementary crystallographic information

### Comment

Transition metal Schiff-base complexes have been intensively studied owing to their interesting physical and chemical properties, including magnetic, optics and catalysis (Ward *et al.*). Herein, we report a crystal structure of an Mn^II^ complex incorporating the Schiff-base ligand, (*E*)-2-(2-hydroxy-3-methoxybenzylideneamino)-2-(hydroxymethyl)propane-\ 1,3-diol.

The asymmetric unit (Fig. 1) comprises one [Mn*L*_2_] complex, two uncoordinated methanol molecules and one lattice water molecule. The coordination geometry of Mn^II^ is distorted octahedral, with the N atoms of the two ligands *trans* to each other. The Mn—O and Mn—N bond distances are in the normal range compared to similar reported complexes (for example, Wang *et al.*, 2007). A complex network of O—H···O hydrogen bonds is formed between the complexes and the lattice methanol and water molecules.

### Experimental

The Schiff-base ligand (H*L*) was synthesized according to the reported literature procedure (Wang *et al.*). The title complex was then prepared by refluxing H*L* (0.050 g, 0.2 mmol) and MnSO_4_.H_2_O in the mixed solvent system CH_3_OH:H_2_O (4:1) until all solid was dissolved. The solution was then cooled to room temperature and filtered. Crystals for diffraction analysis were obtained by slow evaporation of the filtrate. Elemental analysis calculated: C 48.05, H 6.47, N 4.31%; found: C 49.89, H 6.39, N 4.28%.

### Refinement

H atoms bound to C atoms were refined using a riding model with C—H = 0.93 Å, *U*_iso_(H) = 1.2*U*_eq_(C) for aromatic H atoms, C—H = 0.96 Å, *U*_iso_(H) = 1.5*U*_eq_(C) for methyl H atoms and C—H = 0.97 Å, *U*_iso_(H) = 1.2*U*_eq_(C) for methylene H atoms. H atoms bound to most of the O atoms were located in difference Fourier maps and refined with O—H restrained to be 0.85 (1) Å and with *U*_iso_(H) = 1.5*U*_eq_(O). The exceptions were for the disordered C—OH groups and the lattice water molecules: in these cases, the H atoms were placed so as to form reasonable H-bonds with O—H = 0.85 Å and refined as riding with *U*_iso_(H) = 1.5*U*_eq_(O). The C—O bonds of the disordered C—OH groups and the lattice methanol molecules were restrained to a common refined value with an uncertainty of 0.02 %A.

### Crystal data

**Table:**

[Mn(C_12_H_16_NO_5_)_2_]·2CH_4_O·0.5H_2_O	*F*(000) = 1344
*M**_r_* = 636.55	*D*_x_ = 1.420 Mg m^−^^3^
Monoclinic, *P*2_1_/*n*	Mo *K*α radiation, λ = 0.71073 Å
Hall symbol: -P 2yn	Cell parameters from 5287 reflections
*a* = 8.141 (2) Å	θ = 2.0–25.3°
*b* = 18.130 (5) Å	µ = 0.51 mm^−^^1^
*c* = 20.211 (6) Å	*T* = 293 K
β = 93.590 (4)°	Block, pink
*V* = 2977.2 (14) Å^3^	0.12 × 0.10 × 0.09 mm
*Z* = 4	

### Data collection

**Table:**

Bruker APEXII CCD area-detector diffractometer	5287 independent reflections
Radiation source: fine-focus sealed tube	4036 reflections with *I* > 2σ(*I*)
graphite	*R*_int_ = 0.031
φ and ω scans	θ_max_ = 25.3°, θ_min_ = 2.0°
Absorption correction: multi-scan (*SADABS*; Sheldrick, 2003)	*h* = −9→9
*T*_min_ = 0.941, *T*_max_ = 0.955	*k* = −21→17
14576 measured reflections	*l* = −22→24

### Refinement

**Table:**

Refinement on *F*^2^	Primary atom site location: structure-invariant direct methods
Least-squares matrix: full	Secondary atom site location: difference Fourier map
*R*[*F*^2^ > 2σ(*F*^2^)] = 0.051	Hydrogen site location: inferred from neighbouring sites
*wR*(*F*^2^) = 0.148	H atoms treated by a mixture of independent and constrained refinement
*S* = 1.05	*w* = 1/[σ^2^(*F*_o_^2^) + (0.0796*P*)^2^ + 1.8727*P*] where *P* = (*F*_o_^2^ + 2*F*_c_^2^)/3
5287 reflections	(Δ/σ)_max_ = 0.001
416 parameters	Δρ_max_ = 0.93 e Å^−^^3^
16 restraints	Δρ_min_ = −0.65 e Å^−^^3^

### Special details

**Table:**

Geometry. All esds (except the esd in the dihedral angle between two l.s. planes) are estimated using the full covariance matrix. The cell esds are taken into account individually in the estimation of esds in distances, angles and torsion angles; correlations between esds in cell parameters are only used when they are defined by crystal symmetry. An approximate (isotropic) treatment of cell esds is used for estimating esds involving l.s. planes.
Refinement. Refinement of F^2^ against ALL reflections. The weighted R-factor wR and goodness of fit S are based on F^2^, conventional R-factors R are based on F, with F set to zero for negative F^2^. The threshold expression of F^2^ > 2sigma(F^2^) is used only for calculating R-factors(gt) etc. and is not relevant to the choice of reflections for refinement. R-factors based on F^2^ are statistically about twice as large as those based on F, and R- factors based on ALL data will be even larger.

### Fractional atomic coordinates and isotropic or equivalent isotropic displacement parameters (Å^2^)

**Table:**

	*x*	*y*	*z*	*U*_iso_*/*U*_eq_	Occ. (<1)
C1	0.8762 (6)	0.5053 (3)	0.3266 (3)	0.0813 (14)	
H1A	0.9812	0.5090	0.3509	0.122*	
H1B	0.8876	0.5190	0.2813	0.122*	
H1C	0.8368	0.4555	0.3285	0.122*	
C2	0.6105 (4)	0.56122 (19)	0.32411 (18)	0.0476 (8)	
C3	0.5514 (5)	0.5215 (2)	0.26979 (19)	0.0574 (10)	
H3A	0.6184	0.4870	0.2506	0.069*	
C4	0.3910 (6)	0.5330 (2)	0.24340 (18)	0.0604 (10)	
H4A	0.3507	0.5059	0.2068	0.072*	
C5	0.2934 (5)	0.5839 (2)	0.27131 (17)	0.0521 (9)	
H5A	0.1873	0.5919	0.2530	0.062*	
C6	0.3507 (4)	0.62479 (18)	0.32757 (16)	0.0440 (8)	
C7	0.5118 (4)	0.61434 (18)	0.35482 (16)	0.0430 (8)	
C8	0.2333 (4)	0.67685 (19)	0.35280 (17)	0.0450 (8)	
H8A	0.1325	0.6813	0.3288	0.054*	
C9	0.1240 (4)	0.76952 (19)	0.42168 (17)	0.0447 (8)	
C10	0.1292 (4)	0.7749 (2)	0.49750 (18)	0.0524 (9)	
H10A	0.0662	0.8172	0.5106	0.063*	
H10B	0.0813	0.7309	0.5157	0.063*	
C11	−0.0517 (4)	0.7489 (2)	0.3971 (2)	0.0596 (10)	
H11A	−0.1283	0.7852	0.4124	0.071*	
H11B	−0.0608	0.7486	0.3490	0.071*	
C12	0.1665 (5)	0.8444 (2)	0.39232 (19)	0.0563 (9)	
H12B	0.0816	0.8796	0.4021	0.068*	
H12C	0.2694	0.8617	0.4137	0.068*	
C13	0.5914 (9)	1.0165 (3)	0.3854 (3)	0.1014 (18)	
H13A	0.5234	1.0219	0.3452	0.152*	
H13B	0.7017	1.0315	0.3780	0.152*	
H13C	0.5488	1.0467	0.4194	0.152*	
C14	0.6726 (6)	0.9237 (2)	0.46473 (19)	0.0594 (10)	
C15	0.7689 (7)	0.9712 (2)	0.5036 (2)	0.0746 (13)	
H15A	0.7847	1.0194	0.4894	0.090*	
C16	0.8427 (6)	0.9479 (2)	0.5637 (2)	0.0721 (12)	
H16A	0.9086	0.9800	0.5895	0.087*	
C17	0.8178 (5)	0.8778 (2)	0.58459 (19)	0.0547 (9)	
H17A	0.8657	0.8629	0.6254	0.066*	
C18	0.7211 (4)	0.82661 (19)	0.54598 (17)	0.0445 (8)	
C19	0.6493 (4)	0.84878 (19)	0.48343 (16)	0.0428 (8)	
C20	0.7043 (4)	0.75505 (19)	0.57352 (17)	0.0441 (8)	
H20A	0.7661	0.7456	0.6129	0.053*	
C21	0.6069 (5)	0.63052 (19)	0.5864 (2)	0.0567 (10)	
C22	0.4262 (5)	0.6059 (2)	0.5794 (2)	0.0630 (11)	
H22A	0.4184	0.5544	0.5918	0.076*	
H22B	0.3624	0.6346	0.6090	0.076*	
C23	0.6610 (6)	0.6332 (3)	0.6597 (2)	0.0782 (14)	
H23A	0.6497	0.5844	0.6786	0.094*	
H23B	0.7764	0.6468	0.6646	0.094*	
C24	0.7115 (19)	0.5685 (9)	0.5636 (8)	0.076 (4)	0.50
H24A	0.8226	0.5871	0.5609	0.092*	0.50
H24B	0.7156	0.5308	0.5977	0.092*	0.50
O12	0.6698 (8)	0.5379 (3)	0.5091 (3)	0.0742 (17)	0.50
H12	0.6435	0.5701	0.4797	0.111*	0.50
C24A	0.7240 (16)	0.5822 (9)	0.5488 (10)	0.076 (4)	0.50
H24C	0.7127	0.5329	0.5666	0.092*	0.50
H24D	0.6770	0.5803	0.5035	0.092*	0.50
O12A	0.8824 (6)	0.5918 (3)	0.5439 (3)	0.0722 (16)	0.50
H12A	0.8899	0.6168	0.5087	0.108*	0.50
C25	0.5935 (11)	0.8441 (6)	0.2719 (4)	0.169 (4)	
H25A	0.5563	0.8942	0.2672	0.254*	
H25B	0.5871	0.8205	0.2293	0.254*	
H25C	0.7054	0.8436	0.2900	0.254*	
C26	0.2701 (11)	0.8216 (4)	0.7004 (3)	0.131 (3)	
H26A	0.3613	0.8342	0.7307	0.197*	
H26B	0.1866	0.7976	0.7241	0.197*	
H26C	0.2256	0.8656	0.6799	0.197*	
Mn1	0.45435 (5)	0.71273 (2)	0.46924 (2)	0.02874 (16)	
N1	0.2550 (3)	0.71681 (14)	0.40435 (14)	0.0407 (6)	
N2	0.6132 (3)	0.70187 (14)	0.55018 (14)	0.0433 (7)	
O1	0.7639 (3)	0.55283 (16)	0.35476 (15)	0.0694 (8)	
O2	0.5780 (3)	0.65014 (15)	0.40653 (13)	0.0586 (7)	
O3	0.5054 (6)	0.8104 (4)	0.3093 (2)	0.159 (2)	
H3	0.545 (8)	0.814 (5)	0.3492 (7)	0.239*	
O4	0.2975 (3)	0.78214 (14)	0.52223 (12)	0.0496 (6)	
H4	0.297 (6)	0.782 (2)	0.5650 (6)	0.074*	
O5	−0.0915 (3)	0.67810 (19)	0.42156 (19)	0.0826 (10)	
H5	−0.193 (2)	0.668 (3)	0.414 (3)	0.124*	
O6	0.1812 (4)	0.84266 (19)	0.32360 (14)	0.0779 (9)	
H6	0.279 (2)	0.829 (3)	0.316 (3)	0.117*	
O7	0.5916 (5)	0.94260 (17)	0.40552 (15)	0.0847 (10)	
O8	0.5665 (3)	0.80546 (13)	0.44233 (11)	0.0475 (6)	
O9	0.3196 (7)	0.7771 (3)	0.65421 (19)	0.145 (2)	
H9	0.383 (6)	0.7408 (19)	0.664 (5)	0.218*	
O10	0.3606 (3)	0.61528 (15)	0.51303 (15)	0.0656 (8)	
H10	0.294 (5)	0.590 (2)	0.488 (2)	0.098*	
O11	0.5671 (5)	0.6847 (2)	0.69558 (16)	0.0911 (10)	
H11	0.591 (8)	0.679 (4)	0.7369 (9)	0.137*	
O1W	0.0648 (7)	0.5679 (4)	0.4852 (4)	0.0806 (18)	0.50
H1W	0.0147	0.6032	0.4650	0.121*	0.50
H2W	0.0795	0.5224	0.4770	0.121*	0.50

### Atomic displacement parameters (Å^2^)

**Table:**

	*U*^11^	*U*^22^	*U*^33^	*U*^12^	*U*^13^	*U*^23^
C1	0.068 (3)	0.080 (3)	0.097 (4)	0.026 (2)	0.023 (3)	−0.006 (3)
C2	0.048 (2)	0.0451 (19)	0.050 (2)	−0.0014 (16)	0.0098 (15)	−0.0001 (16)
C3	0.078 (3)	0.046 (2)	0.049 (2)	0.0068 (19)	0.0133 (19)	−0.0072 (16)
C4	0.090 (3)	0.052 (2)	0.038 (2)	0.002 (2)	−0.0006 (19)	−0.0120 (16)
C5	0.064 (2)	0.051 (2)	0.0411 (19)	−0.0031 (17)	−0.0026 (16)	−0.0061 (16)
C6	0.0481 (19)	0.0430 (18)	0.0411 (18)	−0.0053 (15)	0.0042 (14)	−0.0057 (14)
C7	0.0434 (18)	0.0434 (18)	0.0429 (18)	−0.0056 (14)	0.0078 (14)	−0.0078 (14)
C8	0.0393 (18)	0.050 (2)	0.0450 (19)	−0.0023 (15)	−0.0040 (14)	−0.0103 (16)
C9	0.0371 (17)	0.0474 (19)	0.049 (2)	0.0034 (14)	−0.0014 (14)	−0.0109 (15)
C10	0.0397 (18)	0.065 (2)	0.053 (2)	0.0014 (16)	0.0040 (15)	−0.0099 (17)
C11	0.0356 (19)	0.071 (3)	0.072 (3)	0.0055 (18)	−0.0029 (17)	−0.018 (2)
C12	0.062 (2)	0.049 (2)	0.056 (2)	0.0033 (18)	−0.0075 (18)	−0.0038 (17)
C13	0.143 (5)	0.072 (3)	0.091 (4)	0.009 (3)	0.019 (4)	0.032 (3)
C14	0.082 (3)	0.049 (2)	0.048 (2)	−0.010 (2)	0.0109 (19)	0.0011 (17)
C15	0.115 (4)	0.047 (2)	0.063 (3)	−0.026 (2)	0.009 (3)	−0.0032 (19)
C16	0.096 (3)	0.055 (2)	0.065 (3)	−0.032 (2)	0.002 (2)	−0.018 (2)
C17	0.063 (2)	0.053 (2)	0.048 (2)	−0.0133 (18)	−0.0015 (17)	−0.0143 (17)
C18	0.0418 (18)	0.047 (2)	0.0447 (19)	−0.0047 (15)	0.0031 (14)	−0.0075 (15)
C19	0.0405 (17)	0.0482 (19)	0.0404 (18)	−0.0071 (15)	0.0072 (14)	−0.0087 (15)
C20	0.0417 (18)	0.0465 (19)	0.0434 (18)	−0.0017 (15)	−0.0040 (14)	−0.0038 (15)
C21	0.053 (2)	0.0377 (19)	0.078 (3)	−0.0042 (16)	−0.0116 (19)	0.0085 (18)
C22	0.058 (2)	0.053 (2)	0.078 (3)	−0.0117 (19)	−0.007 (2)	0.013 (2)
C23	0.075 (3)	0.065 (3)	0.091 (3)	−0.010 (2)	−0.026 (3)	0.034 (3)
C24	0.055 (3)	0.036 (5)	0.140 (8)	−0.009 (3)	0.016 (4)	0.009 (6)
O12	0.086 (4)	0.059 (4)	0.077 (4)	0.007 (3)	0.000 (3)	−0.006 (3)
C24A	0.055 (3)	0.036 (5)	0.140 (8)	−0.009 (3)	0.016 (4)	0.009 (6)
O12A	0.053 (3)	0.066 (4)	0.096 (5)	0.003 (3)	−0.010 (3)	0.018 (3)
C25	0.160 (8)	0.275 (12)	0.074 (4)	−0.007 (8)	0.019 (5)	0.042 (6)
C26	0.208 (9)	0.096 (5)	0.090 (4)	0.016 (5)	0.006 (5)	0.001 (4)
Mn1	0.0227 (2)	0.0306 (3)	0.0325 (3)	−0.00272 (17)	−0.00254 (16)	−0.00772 (18)
N1	0.0329 (14)	0.0443 (15)	0.0449 (16)	−0.0037 (11)	0.0017 (11)	−0.0078 (12)
N2	0.0392 (15)	0.0403 (15)	0.0499 (16)	−0.0038 (12)	−0.0022 (12)	−0.0013 (12)
O1	0.0516 (16)	0.0741 (19)	0.083 (2)	0.0111 (14)	0.0072 (14)	−0.0217 (15)
O2	0.0378 (13)	0.0666 (16)	0.0705 (17)	−0.0002 (12)	−0.0023 (12)	−0.0337 (14)
O3	0.114 (3)	0.299 (7)	0.064 (3)	−0.080 (4)	−0.003 (2)	0.000 (4)
O4	0.0462 (13)	0.0615 (15)	0.0408 (13)	0.0030 (11)	−0.0002 (11)	−0.0082 (12)
O5	0.0400 (15)	0.088 (2)	0.119 (3)	−0.0172 (16)	−0.0023 (16)	−0.007 (2)
O6	0.089 (2)	0.092 (2)	0.0502 (17)	−0.0103 (19)	−0.0159 (16)	0.0114 (15)
O7	0.128 (3)	0.0641 (19)	0.0599 (19)	−0.0113 (19)	−0.0078 (18)	0.0153 (15)
O8	0.0468 (13)	0.0554 (14)	0.0400 (13)	−0.0114 (11)	0.0000 (10)	−0.0050 (10)
O9	0.159 (4)	0.228 (6)	0.047 (2)	0.108 (4)	−0.002 (2)	−0.008 (3)
O10	0.0547 (16)	0.0536 (17)	0.085 (2)	−0.0118 (13)	−0.0213 (14)	0.0063 (14)
O11	0.098 (3)	0.118 (3)	0.0554 (19)	0.015 (2)	−0.0102 (18)	0.020 (2)
O1W	0.050 (3)	0.067 (4)	0.123 (5)	−0.010 (3)	0.000 (3)	−0.010 (4)

### Geometric parameters (Å, °)

**Table:**

C1—O1	1.402 (5)	C19—O8	1.300 (4)
C1—H1A	0.960	C20—N2	1.288 (4)
C1—H1B	0.960	C20—H20A	0.930
C1—H1C	0.960	C21—N2	1.488 (4)
C2—O1	1.367 (5)	C21—C24	1.500 (10)
C2—C3	1.374 (5)	C21—C23	1.521 (6)
C2—C7	1.422 (5)	C21—C24A	1.531 (9)
C3—C4	1.395 (6)	C21—C22	1.535 (5)
C3—H3A	0.930	C22—O10	1.422 (5)
C4—C5	1.363 (5)	C22—H22A	0.970
C4—H4A	0.930	C22—H22B	0.970
C5—C6	1.412 (5)	C23—O11	1.432 (7)
C5—H5A	0.930	C23—H23A	0.970
C6—C7	1.403 (5)	C23—H23B	0.970
C6—C8	1.458 (5)	C24—O12	1.261 (13)
C7—O2	1.316 (4)	C24—H24A	0.970
C8—N1	1.272 (4)	C24—H24B	0.970
C8—H8A	0.930	O12—H12	0.850
C9—N1	1.490 (4)	C24A—O12A	1.311 (12)
C9—C12	1.530 (5)	C24A—H24C	0.970
C9—C11	1.531 (5)	C24A—H24D	0.970
C9—C10	1.533 (5)	O12A—H12A	0.850
C10—O4	1.435 (4)	C25—O3	1.234 (8)
C10—H10A	0.970	C25—H25A	0.960
C10—H10B	0.970	C25—H25B	0.960
C11—O5	1.421 (5)	C25—H25C	0.960
C11—H11A	0.970	C26—O9	1.316 (7)
C11—H11B	0.970	C26—H26A	0.960
C12—O6	1.402 (5)	C26—H26B	0.960
C12—H12B	0.970	C26—H26C	0.960
C12—H12C	0.970	Mn1—O8	2.005 (2)
C13—O7	1.400 (5)	Mn1—O2	2.017 (2)
C13—H13A	0.960	Mn1—N1	2.023 (3)
C13—H13B	0.960	Mn1—N2	2.030 (3)
C13—H13C	0.960	Mn1—O4	2.129 (2)
C14—O7	1.373 (5)	Mn1—O10	2.138 (3)
C14—C15	1.377 (6)	O3—H3	0.86 (1)
C14—C19	1.426 (5)	O4—H4	0.87 (1)
C15—C16	1.388 (6)	O5—H5	0.85 (1)
C15—H15A	0.930	O6—H6	0.86 (1)
C16—C17	1.358 (6)	O9—H9	0.85 (1)
C16—H16A	0.930	O10—H10	0.85 (1)
C17—C18	1.419 (5)	O11—H11	0.85 (1)
C17—H17A	0.930	O1W—H1W	0.850
C18—C19	1.417 (5)	O1W—H2W	0.850
C18—C20	1.422 (5)		
			
O1—C1—H1A	109.5	N2—C21—C24A	102.2 (8)
O1—C1—H1B	109.5	C23—C21—C24A	110.5 (9)
H1A—C1—H1B	109.5	N2—C21—C22	105.7 (3)
O1—C1—H1C	109.5	C24—C21—C22	108.2 (7)
H1A—C1—H1C	109.5	C23—C21—C22	108.5 (4)
H1B—C1—H1C	109.5	C24A—C21—C22	114.2 (7)
O1—C2—C3	124.6 (3)	O10—C22—C21	110.7 (3)
O1—C2—C7	113.8 (3)	O10—C22—H22A	109.5
C3—C2—C7	121.6 (3)	C21—C22—H22A	109.5
C2—C3—C4	120.0 (3)	O10—C22—H22B	109.5
C2—C3—H3A	120.0	C21—C22—H22B	109.5
C4—C3—H3A	120.0	H22A—C22—H22B	108.1
C5—C4—C3	119.9 (3)	O11—C23—C21	112.5 (3)
C5—C4—H4A	120.0	O11—C23—H23A	109.1
C3—C4—H4A	120.0	C21—C23—H23A	109.1
C4—C5—C6	121.1 (4)	O11—C23—H23B	109.1
C4—C5—H5A	119.4	C21—C23—H23B	109.1
C6—C5—H5A	119.4	H23A—C23—H23B	107.8
C7—C6—C5	119.9 (3)	O12—C24—C21	118.1 (12)
C7—C6—C8	124.6 (3)	O12—C24—H24A	107.8
C5—C6—C8	115.5 (3)	C21—C24—H24A	107.8
O2—C7—C6	124.8 (3)	O12—C24—H24B	107.8
O2—C7—C2	117.8 (3)	C21—C24—H24B	107.8
C6—C7—C2	117.4 (3)	H24A—C24—H24B	107.1
N1—C8—C6	126.5 (3)	C24—O12—H12	110.5
N1—C8—H8A	116.8	O12A—C24A—C21	127.4 (11)
C6—C8—H8A	116.8	O12A—C24A—H24C	105.5
N1—C9—C12	107.2 (3)	C21—C24A—H24C	105.5
N1—C9—C11	115.8 (3)	O12A—C24A—H24D	105.5
C12—C9—C11	108.7 (3)	C21—C24A—H24D	105.5
N1—C9—C10	107.5 (3)	H24C—C24A—H24D	106.0
C12—C9—C10	109.8 (3)	C24A—O12A—H12A	104.8
C11—C9—C10	107.8 (3)	O3—C25—H25A	109.7
O4—C10—C9	108.7 (3)	O3—C25—H25B	109.1
O4—C10—H10A	110.0	H25A—C25—H25B	109.5
C9—C10—H10A	110.0	O3—C25—H25C	109.7
O4—C10—H10B	110.0	H25A—C25—H25C	109.5
C9—C10—H10B	110.0	H25B—C25—H25C	109.5
H10A—C10—H10B	108.3	O9—C26—H26A	109.8
O5—C11—C9	109.7 (3)	O9—C26—H26B	109.5
O5—C11—H11A	109.7	H26A—C26—H26B	109.5
C9—C11—H11A	109.7	O9—C26—H26C	109.1
O5—C11—H11B	109.7	H26A—C26—H26C	109.5
C9—C11—H11B	109.7	H26B—C26—H26C	109.5
H11A—C11—H11B	108.2	O8—Mn1—O2	92.73 (11)
O6—C12—C9	113.5 (3)	O8—Mn1—N1	98.86 (10)
O6—C12—H12B	108.9	O2—Mn1—N1	91.42 (10)
C9—C12—H12B	108.9	O8—Mn1—N2	91.25 (10)
O6—C12—H12C	108.9	O2—Mn1—N2	97.70 (11)
C9—C12—H12C	108.9	N1—Mn1—N2	166.06 (11)
H12B—C12—H12C	107.7	O8—Mn1—O4	86.48 (10)
O7—C13—H13A	109.5	O2—Mn1—O4	170.91 (10)
O7—C13—H13B	109.5	N1—Mn1—O4	79.77 (10)
H13A—C13—H13B	109.5	N2—Mn1—O4	91.37 (11)
O7—C13—H13C	109.5	O8—Mn1—O10	170.32 (10)
H13A—C13—H13C	109.5	O2—Mn1—O10	90.02 (12)
H13B—C13—H13C	109.5	N1—Mn1—O10	90.34 (10)
O7—C14—C15	124.5 (4)	N2—Mn1—O10	79.18 (10)
O7—C14—C19	113.8 (3)	O4—Mn1—O10	92.25 (11)
C15—C14—C19	121.7 (4)	C8—N1—C9	119.5 (3)
C14—C15—C16	120.6 (4)	C8—N1—Mn1	125.1 (2)
C14—C15—H15A	119.7	C9—N1—Mn1	115.3 (2)
C16—C15—H15A	119.7	C20—N2—C21	120.5 (3)
C17—C16—C15	119.5 (4)	C20—N2—Mn1	123.1 (2)
C17—C16—H16A	120.2	C21—N2—Mn1	116.1 (2)
C15—C16—H16A	120.2	C2—O1—C1	119.0 (3)
C16—C17—C18	121.9 (4)	C7—O2—Mn1	125.5 (2)
C16—C17—H17A	119.0	C25—O3—H3	110 (3)
C18—C17—H17A	119.0	C10—O4—Mn1	111.1 (2)
C19—C18—C17	119.3 (3)	C10—O4—H4	107 (3)
C19—C18—C20	124.3 (3)	Mn1—O4—H4	123 (3)
C17—C18—C20	116.4 (3)	C11—O5—H5	112 (4)
O8—C19—C18	124.5 (3)	C12—O6—H6	109 (4)
O8—C19—C14	118.6 (3)	C14—O7—C13	118.9 (4)
C18—C19—C14	116.9 (3)	C19—O8—Mn1	124.0 (2)
N2—C20—C18	127.4 (3)	C26—O9—H9	121 (7)
N2—C20—H20A	116.3	C22—O10—Mn1	111.5 (2)
C18—C20—H20A	116.3	C22—O10—H10	133 (4)
N2—C21—C24	117.6 (8)	Mn1—O10—H10	115 (4)
N2—C21—C23	115.8 (3)	C23—O11—H11	109 (5)
C24—C21—C23	100.8 (7)	H1W—O1W—H2W	135.0
			
O1—C2—C3—C4	178.2 (4)	C12—C9—N1—Mn1	87.7 (3)
C7—C2—C3—C4	0.1 (6)	C11—C9—N1—Mn1	−150.8 (3)
C2—C3—C4—C5	0.4 (6)	C10—C9—N1—Mn1	−30.2 (3)
C3—C4—C5—C6	−1.1 (6)	O8—Mn1—N1—C8	105.0 (3)
C4—C5—C6—C7	1.3 (6)	O2—Mn1—N1—C8	12.0 (3)
C4—C5—C6—C8	−179.4 (3)	N2—Mn1—N1—C8	−119.0 (5)
C5—C6—C7—O2	179.1 (3)	O4—Mn1—N1—C8	−170.3 (3)
C8—C6—C7—O2	−0.1 (6)	O10—Mn1—N1—C8	−78.0 (3)
C5—C6—C7—C2	−0.8 (5)	O8—Mn1—N1—C9	−77.4 (2)
C8—C6—C7—C2	180.0 (3)	O2—Mn1—N1—C9	−170.4 (2)
O1—C2—C7—O2	1.9 (5)	N2—Mn1—N1—C9	58.6 (5)
C3—C2—C7—O2	−179.8 (3)	O4—Mn1—N1—C9	7.4 (2)
O1—C2—C7—C6	−178.2 (3)	O10—Mn1—N1—C9	99.6 (2)
C3—C2—C7—C6	0.1 (5)	C18—C20—N2—C21	−178.5 (3)
C7—C6—C8—N1	−4.2 (6)	C18—C20—N2—Mn1	−5.1 (5)
C5—C6—C8—N1	176.6 (3)	C24—C21—N2—C20	−98.6 (8)
N1—C9—C10—O4	45.2 (4)	C23—C21—N2—C20	20.6 (5)
C12—C9—C10—O4	−71.1 (4)	C24A—C21—N2—C20	−99.6 (8)
C11—C9—C10—O4	170.7 (3)	C22—C21—N2—C20	140.6 (3)
N1—C9—C11—O5	56.9 (4)	C24—C21—N2—Mn1	87.6 (8)
C12—C9—C11—O5	177.6 (3)	C23—C21—N2—Mn1	−153.2 (3)
C10—C9—C11—O5	−63.4 (4)	C24A—C21—N2—Mn1	86.6 (7)
N1—C9—C12—O6	57.4 (4)	C22—C21—N2—Mn1	−33.2 (4)
C11—C9—C12—O6	−68.5 (4)	O8—Mn1—N2—C20	16.8 (3)
C10—C9—C12—O6	173.8 (3)	O2—Mn1—N2—C20	109.7 (3)
O7—C14—C15—C16	178.2 (5)	N1—Mn1—N2—C20	−119.9 (4)
C19—C14—C15—C16	−2.1 (7)	O4—Mn1—N2—C20	−69.7 (3)
C14—C15—C16—C17	−0.7 (8)	O10—Mn1—N2—C20	−161.8 (3)
C15—C16—C17—C18	1.4 (7)	O8—Mn1—N2—C21	−169.6 (3)
C16—C17—C18—C19	0.6 (6)	O2—Mn1—N2—C21	−76.7 (3)
C16—C17—C18—C20	−179.8 (4)	N1—Mn1—N2—C21	53.8 (6)
C17—C18—C19—O8	175.9 (3)	O4—Mn1—N2—C21	103.9 (3)
C20—C18—C19—O8	−3.6 (5)	O10—Mn1—N2—C21	11.9 (3)
C17—C18—C19—C14	−3.2 (5)	C3—C2—O1—C1	5.7 (6)
C20—C18—C19—C14	177.3 (3)	C7—C2—O1—C1	−176.1 (4)
O7—C14—C19—O8	4.6 (5)	C6—C7—O2—Mn1	12.7 (5)
C15—C14—C19—O8	−175.2 (4)	C2—C7—O2—Mn1	−167.4 (2)
O7—C14—C19—C18	−176.3 (3)	O8—Mn1—O2—C7	−114.8 (3)
C15—C14—C19—C18	4.0 (6)	N1—Mn1—O2—C7	−15.9 (3)
C19—C18—C20—N2	−6.2 (6)	N2—Mn1—O2—C7	153.6 (3)
C17—C18—C20—N2	174.2 (3)	O10—Mn1—O2—C7	74.5 (3)
N2—C21—C22—O10	44.7 (4)	C9—C10—O4—Mn1	−40.2 (3)
C24—C21—C22—O10	−82.1 (8)	O8—Mn1—O4—C10	118.6 (2)
C23—C21—C22—O10	169.4 (3)	N1—Mn1—O4—C10	19.0 (2)
C24A—C21—C22—O10	−66.9 (10)	N2—Mn1—O4—C10	−150.2 (2)
N2—C21—C23—O11	57.4 (5)	O10—Mn1—O4—C10	−71.0 (2)
C24—C21—C23—O11	−174.6 (8)	C15—C14—O7—C13	−6.8 (7)
C24A—C21—C23—O11	173.0 (7)	C19—C14—O7—C13	173.5 (4)
C22—C21—C23—O11	−61.1 (4)	C18—C19—O8—Mn1	23.1 (5)
N2—C21—C24—O12	−71.5 (16)	C14—C19—O8—Mn1	−157.7 (3)
C23—C21—C24—O12	161.8 (13)	O2—Mn1—O8—C19	−123.4 (3)
C22—C21—C24—O12	48.1 (16)	N1—Mn1—O8—C19	144.7 (3)
N2—C21—C24A—O12A	64 (2)	N2—Mn1—O8—C19	−25.6 (3)
C23—C21—C24A—O12A	−60 (2)	O4—Mn1—O8—C19	65.7 (3)
C22—C21—C24A—O12A	177.2 (15)	C21—C22—O10—Mn1	−36.8 (4)
C6—C8—N1—C9	177.6 (3)	O2—Mn1—O10—C22	112.2 (3)
C6—C8—N1—Mn1	−4.9 (5)	N1—Mn1—O10—C22	−156.3 (3)
C12—C9—N1—C8	−94.5 (4)	N2—Mn1—O10—C22	14.4 (3)
C11—C9—N1—C8	26.9 (5)	O4—Mn1—O10—C22	−76.6 (3)
C10—C9—N1—C8	147.5 (3)		

### Hydrogen-bond geometry (Å, °)

**Table:**

*D*—H···*A*	*D*—H	H···*A*	*D*···*A*	*D*—H···*A*
O3—H3···O8	0.86 (1)	1.89 (1)	2.706 (5)	160 (4)
O4—H4···O9	0.86 (1)	1.80 (1)	2.664 (5)	174 (5)
O5—H5···O2^i^	0.85 (1)	1.89 (1)	2.736 (4)	173 (6)
O6—H6···O3	0.86 (1)	1.89 (1)	2.737 (7)	172 (6)
O9—H9···O11	0.85 (1)	1.89 (1)	2.712 (5)	162 (4)
O10—H10···O1W	0.86 (1)	1.90 (4)	2.585 (6)	136 (5)
O11—H11···O6^ii^	0.85 (1)	1.90 (2)	2.739 (4)	169 (7)
O12—H12···O2	0.85	2.12	2.967 (7)	180
O12A—H12A···O5^iii^	0.85	2.10	2.946 (7)	180
O1W—H1W···O5	0.85	1.81	2.657 (8)	180
O1W—H2W···O12A^iv^	0.85	2.14	2.990 (9)	180

Symmetry codes: (i) *x*−1, *y*, *z*; (ii) *x*+1/2, −*y*+3/2, *z*+1/2; (iii) *x*+1, *y*, *z*; (iv) −*x*+1, −*y*+1, −*z*+1.
